# Effectiveness of community-based integrated care in frail COPD patients: a randomised controlled trial

**DOI:** 10.1038/npjpcrm.2015.22

**Published:** 2015-04-09

**Authors:** Carme Hernández, Albert Alonso, Judith Garcia-Aymerich, Ignasi Serra, Dolors Marti, Robert Rodriguez-Roisin, Georgia Narsavage, Maria Carmen Gomez, Josep Roca, NEXES consortium

**Affiliations:** 1 Medical and Nursing Direction, Hospital Clinic de Barcelona. CIBER en Enfermedades Respiratorias (CIBERES), Institut d’Investigacions Biomèdiques August Pi i Sunyer (IDIBAPS), Universitat de Barcelona, Barcelona, Spain; 2 Information System Department, Hospital Clinic de Barcelona, CIBER en Enfermedades Respiratorias (CIBERES), Institut d’Investigacions Biomèdiques August Pi i Sunyer (IDIBAPS), Universitat de Barcelona, Barcelona, Spain; 3 Centre for Research in Environmental Epidemiology (CREAL), CIBER Epidemiologia y Salud Pública (CIBERESP), Department of Experimental and Health Sciences, Universitat Pompeu Fabra, Barcelona, Spain; 4 Programa d’Atenció Domiciliaria i Equips de Suport (PADES), Grup MUTUAM, Barcelona, Spain; 5 Interprofessional Education, West Virginia University, Morgantown, WV, USA; 6 Nursing Direction, Hospital Clinic, Universitat de Barcelona, Barcelona, Spain

## Abstract

**Background:**

Chronic obstructive pulmonary disease (COPD) generates a high burden on health care, and hospital admissions represent a substantial proportion of the overall costs of the disease. Integrated care (IC) has shown efficacy to reduce hospitalisations in COPD patients at a pilot level. Deployment strategies for IC services require assessment of effectiveness at the health care system level.

**Aims:**

The aim of this study was to explore the effectiveness of a community-based IC service in preventing hospitalisations and emergency department (ED) visits in stable frail COPD patients.

**Methods:**

From April to December 2005, 155 frail community-dwelling COPD patients were randomly allocated either to IC (*n*=76, age 73 (8) years, forced expiratory volume during the first second, FEV_1_ 41(19) % predicted) or usual care (*n*=84, age 75(9) years, FEV_1_ 44 (20) % predicted) and followed up for 12 months. The IC intervention consisted of the following: (a) patient’s empowerment for self-management; (b) an individualised care plan; (c) access to a call centre; and (d) coordination between the levels of care. Thereafter, hospital admissions, ED visits and mortality were monitored for 6 years.

**Results:**

IC enhanced self-management (*P*=0.02), reduced anxiety–depression (*P*=0.001) and improved health-related quality of life (*P*=0.02). IC reduced both ED visits (*P*=0.02) and mortality (*P*=0.03) but not hospital admission. No differences between the two groups were seen after 6 years.

**Conclusion:**

The intervention improved clinical outcomes including survival and decreased the ED visits, but it did not reduce hospital admissions. The study facilitated the identification of two key requirements for adoption of IC services in the community: appropriate risk stratification of patients, and preparation of the community-based work force.

## Introduction

Chronic obstructive pulmonary disease (COPD) has a high impact on health care use and hospital admissions^1,2^ represent a marked proportion of the overall costs of the disease.^3–6^ Age, co-morbidities and previous history of multiple hospital admissions are well identified as major frailty factors with a high risk for unplanned admissions that are often associated with accelerated disease progression.^7,8^

Conventional health care is too often oriented towards solving acute events with a disease-oriented approach that has been proven to be suboptimal for chronic patients.^7,9^ Consequently, the deployment of effective and efficient health care models for patients with complex chronic conditions^10^ following an integrated care (IC) approach^11–13^ is a widely accepted priority for improving health systems. However, studies exploring long-term outcomes and related factors that may limit extensive deployment and sustainability of IC services are still needed.^10,14–16^

The literature on effectiveness of integrated disease management in COPD patients displays controversial results. A recent Cochrane review^15^ acknowledges high variability^16^ among well-conceived randomised controlled trials (RCTs) on IC interventions, but it concludes that IC shows positive outcomes in terms of enhanced health-related quality of life, exercise capacity and reduction of hospital requirements with no differences in mortality rate. These results are consistent with a high potential for cost reduction.^17^ However, there is a need for additional RCTs with well-standardised interventions providing contextual information on the characteristics of the health care systems wherein IC is deployed to facilitate comparability among studies.

In a previously reported RCT using IC services for care of COPD patients,^18^ an IC intervention run by hospital-based specialised nurses reduced hospitalisations due to severe exacerbations by 40%. The patients were recruited from two tertiary hospitals (Barcelona, Spain and Leuven, Belgium) immediately after discharge for an exacerbation of their respiratory disease, with no differences in health care outcomes between the two sites despite the differences in the two national health care systems.

The principal objective of the current research was to assess the effectiveness of the IC intervention^18^ in frail COPD patients representative of a high-risk subset of patients in the population. The main difference with Casas *et al.* ^18^ was that the present study delivered an IC service with a community-based approach using a distributed model. That is, patients were recruited without major restrictions regarding co-morbidities, and hospital-based respiratory nurse specialists played a supporting role to community-based care professionals.

This study was aimed at analysing the potential for extensive deployment of the previously reported IC intervention^18^ by introducing five new elements: (a) clinically stable, albeit frail, COPD patients selected from the community clinics rather than recruited after hospital discharge; (b) a patient-centred inclusive approach, with COPD as one diagnosis with higher severity and more co-morbid conditions; (c) very low support by the specialised team; (d) inclusion of community-based teams in the IC intervention; and (e) two different providers. The IC intervention was provided for 12 consecutive months, and then patients were followed up passively for 6 additional years.

## Materials and methods

### Study group

The research was designed to explore the potential for generalisation of the IC service reported by Casas *et al.* ^18^ and provide relevant preliminary information for the preparatory phase of the NEXES project (2008–13).^11^ The NEXES project has successfully explored strategies for extensive deployment of four different types of IC services covering the entire spectrum of severity of chronic patients. One of these four services was enhanced care for frail chronic patients to prevent hospital admissions. The current manuscript reports on the lessons learned for deployment of this community-based IC service, following patients for 6 years beyond the NEXES^19,20^ project.

A total of 155 clinically stable COPD patients with a history of at least two hospital admissions owing to severe respiratory exacerbations during two consecutive years were included in the NEXES study from April to December 2005. At the time of the current study, 140 of these patients had been managed under a conventional treatment regime (naïve patients), and the remaining 15 had been enrolled in the hospital’s specialist nurse-based enhanced care programme for COPD patients^18^ (Note that these 15 patients were statistically excluded in the final analyses). The recruitment was done using the records of our Institution (Hospital Clínic at Barcelona-Esquerra). All patients signed the informed consent after full explanation of the characteristics of the protocol. Ethics approval was granted by the Ethical Committee of the Hospital Clinic.

In the selection process for the community-based study, we considered a broad spectrum of COPD diagnostic terms that include chronic obstructive inflammatory diseases namely, emphysema, asthma, tuberculosis, chronic bronchitis and COPD. Other inclusion criteria were aged above 45 years and living at home within the health care area of the hospital (Barcelona-Esquerra).

**Figure 1:**
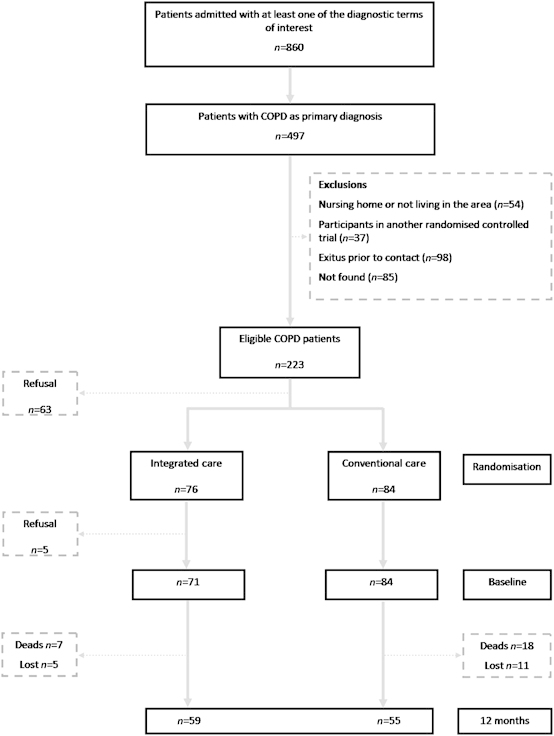
Flowchart of the study.

A total of 2,454 episodes of hospital admission corresponding to 860 chronic respiratory patients were identified using the inclusion criteria. From this population, a person not involved in the study identified the cases with COPD (ICD9-CM 491, 492, 493 or 496)^21^ as the primary diagnosis for admission. Figure 1 displays the main exclusion criteria in the 160 patients eligible for the study. A computer-generated list of random numbers with no restrictions and administered by personnel who were not involved in the study ensured blinded randomisation (1:1 ratio); 76 patients were included in the community-based intervention group (IC) and 84 in Usual Care (UC). The intervention was active over 12 months, and the two groups were passively followed up for 6 additional years. Records of the 155 patients who began the study and the 112 patients who completed the 1-year follow-up were identified for review at 6 years.

### Integrated care

The intervention included four key features. (1) A comprehensive assessment of the patient at entry, including severity of the respiratory disease, evaluation of co-morbid conditions and analysis of social support needs, was completed.^22^ (2) A 2-h educational programme was administered at entry by a respiratory nurse, followed by distribution of patient-specific support material (www.separ.es).^23^ The educational programme covered knowledge of the disease, instructions on non-pharmacological treatment, administration techniques for proper pharmacological therapy and techniques for self-management of the disease and co-morbid conditions including strategies to adopt with future exacerbations. (3) One joint visit of the specialised nurse and the primary care team (physician, nurse and social worker) at the patient home was completed within 72 h after entry into the study. During this visit, the therapeutic plan for each patient was customised to their individual frailty factors^13,18,24,25^ and shared with the primary care team. Reinforcement of the logistics for treatment of co-morbidities and social support was done accordingly. (4) Accessibility to the specialised nurse at the hospital was ensured for primary care professionals during the follow-up period through an ICT platform^18,26–28^ including a web-based call centre.

The community care teams received training: a 2-h face to face educational training and 1-day stay at the hospital ward, aiming at enhancing home-based management of frail COPD patients. The number of home care visits during the 12-m follow-up period was individually tailored to patient needs. Moreover, planned visits by respiratory professionals were scheduled through the day hospital or home visits if this was deemed necessary by primary care teams.

### Usual care

Patients assigned to UC followed conventional treatment, being managed by their physician without any support from specialised nurses. Visits were usually scheduled every 6 months in the out-patient clinic.

### Assessment

A blind evaluation of the study group carried out before randomisation and after the 12-month follow-up consisted of a patient interview and analysis of medical records, self-administered questionnaires and lung function testing. The questionnaires were those used by Casas *et al.* ^18^ with additions designed to capture patient empowerment characteristics: mental status,^29^ activities of daily living,^30,31^ anxiety and depression,^32^ health-related quality of life,^33^ Epworth sleepiness scale,^34,35^ 6-min walking test,^36^ nocturnal pulse oximetry and measurement of body mass distribution using bioelectric impedance.^37^ Exacerbations requiring emergency department (ED) consultations and/or hospital admissions within the health care sector (Barcelona-Esquerra, 540.000 inhabitants) were assessed using shared registries from the public health system. The amount of potentially missed episodes of severe exacerbations owing to the attendance of patients by private health care providers is negligible.

Six years after the end of the study, medical records and mortality were reviewed in all patients.

### Statistical analysis

Sample size calculation^38^ was obtained considering an IC:UC ratio of 1:1. Estimating a proportion of 60% admitted in the UC group,^18^ accepting an alpha risk of 0.05 and a beta risk of 0.20 in a two-sided test and anticipating a drop-out rate of 0.15, a sample size of 73 patients per group was necessary to recognise a statistically significant relative risk of re-admission of at least 0.70 in the IC versus the UC groups. This magnitude of effect is lower than the relative risk of readmission (RR) (IC versus UC=0.55) previously observed.^18^ The number of patients identified during the screening process (*n*=155) was higher than the 146 required, thus ensuring enough statistical power.

Results are expressed as mean and s.d. or as number and percentages in the corresponding categories. Comparisons between the two study groups on admission and at 12 months were performed using unpaired Student's *t*-test for continuous variables and *Χ* ^2^-tests for non-continuous variables. Changes within each group were assessed by paired analysis. The effects of the intervention on the rate of admissions and mortality were analysed, respectively, using multivariate logistic and Cox regression analysis, adjusted for baseline differences between groups. Qualitative assessment of technology was done using standard questionnaires and through focus groups. As a sensitivity analysis, we repeated all analyses after excluding patients who had a previous contact with any IC programme (*n*=15). Statistical significance was set at a *P* value <0.05. Analyses were done with STATA release 10.0 (2008, StataCorp LP, College Station, TX, USA).

## Results

### Characteristics of the study group

At entry (Table 1, *n*=155), the patients of the two groups showed similar characteristics, except that influenza and pneumococcal vaccination were more prevalent in the IC group (*P*<0.01). Patients in both IC and UC groups were elderly, 14% were current smokers defined as ‘smoke every day or some days’ and 35% had an education level lower than primary school completion. It is noteworthy that 55% were aware of the name of their COPD disease, and 70% could identify an episode of exacerbation. The average number of co-morbidities per patient was six. No significant differences between arms were observed in number and categories of co-morbidities, previous history of emergency room visits and hospital admissions.

As indicated in Figure 1, 25 patients (22%) died and 16 (14%) were lost during the 1-year follow-up, and the remaining 114 patients (IC, *n*=59 and UC, *n*=55) completed the 12-month assessment visit. Patients lost to follow-up exhibited a higher affectation of activities of daily living and health-related quality of life at baseline than those who were followed up, without differences in other sociodemographic, clinical, functional and medical care-related variables.

### Effects after 12-month IC intervention

All 155 enrolled patients were included in this intention-to-treat analysis. After adjusting for baseline differences, the IC arm strongly reduced both the risk of emergency room visits (*P*=0.02) and mortality (*P*=0.03), as displayed in Table 2. No differences between arms were observed in admissions neither owing to respiratory cause nor to other causes. However, the IC intervention markedly changed the pattern of hospitalisations. Eighty percent of the admissions in the IC arm were coordinated (planned) between primary care and the hospital team, thus not using the ED. By contrast, all admissions (100%) in the UC arm were processed as unplanned hospitalisations through the ED (*P* value not computable).

As secondary outcomes, we compared the final characteristics of 114 patients followed up with 12 months between intervention and UC groups. Patients in the IC arm showed a lower percentage of current smokers (UC, 16% vs. ICS, 3%, *P*=0.02), better COPD knowledge and self-management (50% vs. 71%, *P*=0.02), lower depression score (Anxiety and Depression Scale, HADS)^32^ (mean 7 vs. 5, *P*<0.01) and fewer symptoms score in the St George's Respiratory Questionnaire (mean 42 vs. 32, *P*=0.02) than in the UC arm (Table 3).

After the study, patients in the intervention group returned to UC. The assessment carried out after 6 years of passive follow-up did not show any significant difference between the two arms in mortality or hospital admissions. It was not possible to identify the role of planned versus unplanned admissions from the records.

## Discussion

### Main findings

In the current research, we observed that the community-based IC approach significantly enhanced survival in the intervention arm. To our understanding, the study generated three well-defined lessons learned with a high potential for generalisation.

First, a standardised intervention with a holistic approach based on shared agreements across levels of care markedly decreased emergency room visits and completely changed the usual profile of unplanned admissions^7^ towards planned hospitalisations over 1 year of active IC management and coordination. However, after 6 years, no differences between the two groups were seen, which suggests that the approach was not adequately adopted at the community level and thus the intervention was not continued.

Second, after the 1-year follow-up, the treatment group presented evidence of a healthier lifestyle, improved self-management and higher health-related quality of life than the control group. All these achievements were specific to the IC intervention group and are consistent with the significant decrease in mortality rate observed in the intervention group.

The third was a not less important consideration. Despite the similarities of this study outcome with those of the IC intervention by Casas *et al.*,^18^ we were not able to reduce hospital admissions and/or mean hospital stay over time. Among the differences between the two studies, significant factors to be taken into consideration in the interpretation of the longitudinal results is the fact that the role of the specialised team, by study design, was to provide support to the community team at demand. In other words, although the Casas *et al.* study^18^ showed the typical characteristics of a pilot approach wherein a hospital-based specialised nurse takes direct care of all the elements of the intervention, the current investigation is closer to deployment of community-based IC run by primary care professionals.

### Interpretation of findings in relation to previously published work

As inidcated in the Introduction, the literature on effectiveness of integrated disease management in COPD patients displays controversial results. The current research suggests that the organisational component, i.e., the management of the change and, in particular, the preparation of the workforce, emerges as one of the most relevant factors required to ensure successful scalability of this type of IC intervention at a regional level.^10,39–46^ We acknowledge, however, that other differential factors might limit the comparability between the two studies. An additional limiting factor for the current study compared with Casas *et al.* ^18^ as a disease-oriented approach involving stronger exclusion criterion, whereas the current investigation included patients with a higher number of more severe co-morbid conditions, a strategy better aligned with real-world patient-centred care.

Regarding the potential impact of frailty, multiple studies^7,8,47^ clearly indicate that age and co-morbidities are two of the main factors contributing to the patient’s frailty and modulating an individual’s risk profile. As indicated above, patients in the current study presented these two factors (mean age 74 years and a mean of six co-morbidities), which, in turn, may explain the history of previous admissions despite the fact that our patients only had moderate to severe chronic respiratory disease (mean forced expiratory volume during the first second 42% predicted). Other determinants of patient’s frailty are anxiety–depression and fragmentation of care^7^ that can ultimately lead to lower health-related quality of life with an enormous use of resources and poor prognosis. The lack of an operational definition of frailty^48^ and complexity for COPD patients can be seen as a pivotal limiting element precluding the association of specific shared-care agreements with patient stratification based on frailty profiles.

In summary, two key components for addressing the complexities of care include (i) appropriate health risk assessment^49^ (case finding) and subsequent patient stratification and (ii) efficient workflow designs across levels of care including IC services with shared-care agreements between specialised and primary care including social support.^10^ The difficulties of managing the complex care of our patients with multiple chronic conditions can be seen in the (unjustified) scepticism raised by some studies on the role of IC services in COPD patients.^3^

### Strengths and limitations of this study

The manuscript reports data collected in a RCT enriched by the lessons learned during the NEXES lifespan, which, we believe, highly strengthen the conclusions. However, we identified two major issues that could be interpreted as weaknesses of the report. First, although the study was officially registered as part of the EU Grant (CIP-ICT-PSP-2007.225025), the RCT was not included in the clinicaltrials.gov registry because at that time it was not compulsory. The second limitation is the long delay between data collection and reporting, which can be justified by the fact that the current manuscript uses preparatory data to support lessons learnt throughout the project over a 6-year longitudinal record review.

### Implications for future research, policy and practice

The study raises two core issues for the deployment and adoption of community-based IC. The role of change management and workforce preparation are crucial to institutionalisation of a system change. Another challenge is to address, in a dynamic manner, the interplay between population-based stratification for case finding and individualised risk prediction to support health professionals in the decision-making process. There is a clear need to successfully impact both challenges in order to succeed in providing personalised care for chronic patients within an IC system.

### Conclusions

The community-based IC intervention generated positive clinical outcomes in terms of mortality, ED admissions and self-management of COPD patients, but not hospital admissions. However, we conclude that further progress in (a) operational strategies to deal with frailty at individual level and (b) workforce preparation are priority issues that are needed for extensive deployment of the chronic care model. The present study provides insights into unmet needs of a coordinated care approach for the management of frail COPD patients with multi-morbidities.

## Figures and Tables

**Table 1:** Baseline characteristics of the study groups

	*UC*, n*=84*	*IC*, n*=71*	P *values*
*Sociodemographics*			
Age (years), mean (s.d.)	75 (9)	73 (8)	0.21
Gender (female), *n* (%)	12 (14)	12 (17)	0.65
Active smokers, *n* (%)	12 (14)	9 (13)	0.77
			
*Clinical and functional profiles*, *mean (s.d.)*			
FVC (% pred)	67 (20)	62 (19)	0.11
FEV_1_ (% pred)	44 (20)	41 (19)	0.27
FEV_1_/ FVC (%)	0.47 (0.15)	0.47 (0.13)	0.96
PaO_2_ (mm Hg)	68 (13)	69 (10)	0.93
PaCO_2_ (mm Hg)	40 (8)	41 (19)	0.69
BMI (kg/m^2^)	27 (5)	29 (5)	0.12
MRC dyspnoea scale	2.5 (1.3)	2.7 (1.3)	0.38
6MWT ( m)	357 (82)	355 (103)	0.93
Co-morbidities	6 (3)	6 (3)	0.98
Mini-mental scale (MEC; 0–35)	29 (4)	30 (4)	0.06
Lawton Index (0–8)	4.4 (2)	5 (2)	0.50
HADS (0–15)			
Anxiety	6 (5)	6 (4)	0.23
Depression	6 (5)	6 (4)	0.30
Quality of life (SGRQ), total score (0–100)	49 (21)	47 (19)	0.47
Number of previous admissions	1.7 (1.2)	1.8 (1.0)	0.61
			
*COPD treatment,* n *(%)*			
Influenza vaccination	68 (81)	66 (94)	0.01
Pneumococal vaccination	40 (55)	48 (75)	0.01
LTOT	32 (39)	30 (43)	0.64
Long-acting β_2_-agonists	32 (39)	22 (31)	0.30
Anticholinergics	78 (95)	66 (93)	0.57
Inhaled glucocorticosteroids	63 (75)	58 (82)	0.32

Abbreviations: BMI, body mass index; COPD, chronic obstructive pulmonary disease; FEV_1_, forced expiratory volume during the first second; FVC, forced vital capacity; HADS, Hospital Anxiety/Depression Scale; IC, integrated care; Lawton Index, performance in activities of daily living; LTOT, long-term oxygen therapy; MEC, mental status; MRC, Medical Modified Research Council Scale for scoring dyspnoea; 6MWT, 6- min walking distance; PaO_2_ and PaCO_2_, partial pressure of oxygen and carbon dioxide, respectively, breathing room air; SGRQ, Saint George’s Respiratory Questionnaire to assess health-related quality of life; UC, usual care.

Results are expressed either as mean±s.d. or as number (percentage) of subjects in the corresponding category.

**Table 2:** Effects of the integrated care intervention, compared with usual care, in 155 frail COPD patients

	*OR* a *(95% CI)*	P *values*
Hospital admissions owing to COPD exacerbations	2.17 (0.60–7.87)	0.237
Emergency room admissions owing to COPD exacerbations	0.33 (0.13–0.84)	0.020
	*HR* a *(95% CI)*	
All-cause mortality	0.36 (0.14–0.93)	0.034

Abbreviations: CI, confidence interval; COPD, chronic obstructive pulmonary disease; OR, odds ratio.

a Adjusted for baseline differences between usual care and integrated care group (influenza and pneumococcal vaccination).

**Table 3:** Comparison between UC and IC at the end of the 12-month follow-up

	*UC,* n*=55*	*IC*, n*=59*	P *values*
Active smokers, *n* (%)	9 (16)	2 (3)	0.02
MRC dyspnoea scale, mean (s.d.)	2.4 (1.3)	2.4 (1.2)	0.96
Lawton index (0–8), mean (s.d.)	6.2 (0.9)	6.3 (0.8)	0.26
*HADS (0–15)*			
Anxiety, mean (s.d.)	7 (4)	5 (4)	0.13
Depression, mean (s.d.)	7 (5)	5 (3)	<0.01
			
*Quality of life (SGRQ), mean (s.d.)*			
Total score (0–100)	49 (22)	43 (20)	0.13
Symptoms score (0–100)	42 (24)	32 (20)	0.02
Activity score (0–100)	69 (24)	63 (26)	0.20
Impacts score (0–100)	40 (24)	36 (21)	0.28
Influenza vaccination, *n* (%)	47 (86)	57 (98)	0.01
Pneumococcal vaccination, *n* (%)	28 (70)	49 (89)	0.02
LTOT, *n* (%)	28 (51)	39 (66)	0.10
Long-acting β_2_-agonists, *n* (%)	20 (36)	10 (17)	0.02
Anticholinergics, *n* (%)	49 (89)	56 (95)	0.25
Inhaled steroids, *n* (%)	50 (60)	53 (70)	0.18
COPD knowledge and self-management, *n* (%)	25 (50)	40 (71)	0.02

Abbreviations: COPD, chronic obstructive pulmonary disease; HADS, Hospital Anxiety/Depression Scale; IC, integrated care; LTOT, long-term oxygen therapy; MRC, Medical Modified Research Council Scale for scoring dyspnoea; SGRQ, Saint George’s Respiratory Questionnaire to assess health-related quality of life; UC, usual care.

Results are expressed either as mean±s.d. or as number (percentage) of subjects in the corresponding category.
